# Towards 3D Multi-Layer Scaffolds for Periodontal Tissue Engineering Applications: Addressing Manufacturing and Architectural Challenges

**DOI:** 10.3390/polym12102233

**Published:** 2020-09-28

**Authors:** Marta Porta, Chiara Tonda-Turo, Daniele Pierantozzi, Gianluca Ciardelli, Elena Mancuso

**Affiliations:** 1 PolitoBIOMed Lab, Department of Mechanical and Aerospace Engineering, Politecnico di Torino, Corso Duca Degli Abruzzi 29, 10129 Turin, Italy; marta.porta94@gmail.com (M.P.); chiara.tondaturo@polito.it (C.T.-T.); gianluca.ciardelli@polito.it (G.C.); 2 Nanotechnology and Integrated Bio-Engineering Centre (NIBEC), Ulster University, Shore Road, Newtownabbey BT37 0QB, UK; daniele.pierantozzi84@gmail.com

**Keywords:** polymer-based composites, multi-layer scaffold, single-step additive manufacturing, periodontal tissue engineering

## Abstract

Reduced periodontal support, deriving from chronic inflammatory conditions, such as periodontitis, is one of the main causes of tooth loss. The use of dental implants for the replacement of missing teeth has attracted growing interest as a standard procedure in clinical practice. However, adequate bone volume and soft tissue augmentation at the site of the implant are important prerequisites for successful implant positioning as well as proper functional and aesthetic reconstruction of patients. Three-dimensional (3D) scaffolds have greatly contributed to solve most of the challenges that traditional solutions (i.e., autografts, allografts and xenografts) posed. Nevertheless, mimicking the complex architecture and functionality of the periodontal tissue represents still a great challenge. In this study, a porous poly(ε-caprolactone) (PCL) and Sr-doped nano hydroxyapatite (Sr-nHA) with a multi-layer structure was produced via a single-step additive manufacturing (AM) process, as a potential strategy for hard periodontal tissue regeneration. Physicochemical characterization was conducted in order to evaluate the overall scaffold architecture, topography, as well as porosity with respect to the original CAD model. Furthermore, compressive tests were performed to assess the mechanical properties of the resulting multi-layer structure. Finally, in vitro biological performance, in terms of biocompatibility and osteogenic potential, was evaluated by using human osteosarcoma cells. The manufacturing route used in this work revealed a highly versatile method to fabricate 3D multi-layer scaffolds with porosity levels as well as mechanical properties within the range of dentoalveolar bone tissue. Moreover, the single step process allowed the achievement of an excellent integrity among the different layers of the scaffold. In vitro tests suggested the promising role of the ceramic phase within the polymeric matrix towards bone mineralization processes. Overall, the results of this study demonstrate that the approach undertaken may serve as a platform for future advances in 3D multi-layer and patient-specific strategies that may better address complex periodontal tissue defects.

## 1. Introduction

Oral diseases represent a significant health and economic problem, affecting about 3.5 billion people worldwide [1]. This burden is expected to increase as a direct consequence of the aging global population, of which more than 20% will be older than 65 by 2050 [2,3]. Among the different oral conditions, tooth loss has been recognized as one of the most common [4]. This can be the result of trauma, oral cavities, cancer and periodontal diseases, which comprise of periodontitis and gingivitis. Periodontitis is a chronic inflammatory condition, characterized by the damage of the periodontium, which include the hard (i.e., alveolar bone and cementum) and soft (i.e., gingiva and periodontal ligament) tissues that surround the teeth. In case of severe periodontitis, reduced periodontal support can lead to tooth movement and eventually tooth loss [5].

As such, the use of dental implants for the replacement of missing teeth has attracted growing interest as a standard procedure. However, to ensure successful implant placement, the presence of adequate vertical and horizontal bone as well as soft tissue is fundamental [6]. Bone tissue augmentation is a procedure of paramount importance to provide mechanical stability and aesthetic functionality, before dental implant placement into patients. Augmentation can be achieved through the use of different bone grafting materials (including autografts, allografts and xenografts) and with properties that vary from space maintenance, blood clot stabilization and scaffolding, by providing a temporary template to support tissue regeneration [7,8].

Even though bone grafting materials have been a long-standing strategy in the regeneration of dentoalveolar bone tissue, they suffer from several disadvantages that include poor processability, possibility of infections and immune reactions, high costs and long surgery times [9].

Tissue engineering approaches that leverage biomaterials as well as manufacturing technologies have opened new doors for dentoalveolar reconstruction through the introduction of 3D scaffolds [10]. In order to support hard periodontal tissue regeneration, it has been established that a 3D scaffold should be biocompatible and bioactive to allow bone tissue bonding [11], it should have a total porosity similar to human cancellous bone (30–90%) and be characterized by an interconnected network of pores, which should be in the range between 150 and 500 μm, in order to facilitate vascularization and new tissue formation without compromising its overall mechanical stability [12]. Regarding the mechanical performance, the scaffold should possess adequate mechanical strength until full tissue formation is achieved. Moreover, and in order to guarantee its function, the scaffold should degrade at a rate that has to match the bone remodeling process, which in the case of an alveolar bone within 5–6 months has been considered optimal [8,13].

At present, the most widely applied biomaterials for bone scaffolds’ production include synthetic polymers (such as polycaprolactone (PCL), polylactic acid (PLA), polyglycolic acid (PGA) and polylactic-co-glycolic acid (PLGA)) [14,15,16], as well as bioceramics (such as β-tricalcium phosphate (β-TCP), hydroxyapatite (HA) and its doped alternatives) [17,18,19,20]. Polymeric materials have showed great promise as 3D substitutes for bone tissues, particularly for their widely demonstrated biocompatibility, biodegradability and easy processability [21,22]; whereas bioceramics have been mainly investigated for their similarity to the inorganic phase of native bone tissue, and thus for their positive outcomes towards osteointegration, osteoinduction and osteoconduction [23]. However, given the composite characteristics of native human bone, the promising combination of polymers and ceramics, and where each phase contributes with its own strengths to the final biomaterial-based solution, has been recognized by several research works [16,24,25,26].

Within the periodontal tissue engineering field, the benefits in using HA and its doped formulations to promote osteoblast-like cell adhesion, differentiation and proliferation, leading to faster osteointegration and enhanced bone tissue formation has been demonstrated by different research groups [27,28,29,30]. Additionally, the ability of nHA to promote human periodontal ligament cell proliferation as well as its therapeutic effect on periodontal epithelium have also been established [28,31]. Particularly, strontium (Sr) is a promising trace element that, by replacing the calcium in the HA lattice, has shown great ability in supporting new bone formation by inducing osteoblast and preventing osteoclast activity [32,33]. In a recent study from the authors it has been demonstrated that Sr-containing composite scaffolds showed greater levels of mineralization and osteogenic potential in comparison to bare PCL and pure HA-based composites [34]. Furthermore, Tsai et al. reported on the fabrication of PCL membranes containing strontium-doped nano hydroxyapatite (Sr-nHA) nanofibers and their potential for guided bone regeneration applications [35]. However, periodontal regeneration involves a set of complex tissues and structures in and around the tooth; hence, a biomaterial-based approach should involve the use of a functionally graded scaffold, where the architecture as well as the composition of each comportment match the fine organization of the tissue that needs to be regenerated [36,37,38]. In 2015, Rasperini et al. fabricated the first patient-specific additively biomanufactured scaffold for periodontal bone regeneration in humans, by using a PCL/HA composite produced via selective laser sintering technology [39]. Even though Rasperini et al. established the potential of the additively manufactured scaffold for the treatment of a large periodontal bone defects, the long-term results, particularly the limited bone regeneration, suggested the need for incorporating scaffold design imperatives (i.e., larger and interconnected pores network). In the past decade, novel AM technologies, i.e., multi-extrusion 3D printing, have been explored in the whole field of tissue engineering, showing great promise as a valuable strategy for the development of multi-layer scaffolds [40,41]. However, in clinical dentistry this approach is still in its beginning and yet to be marginally investigated.

**Figure 1:**
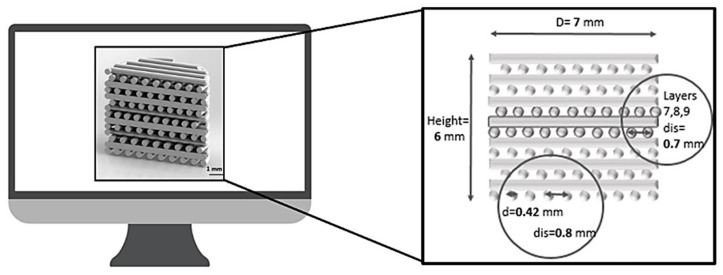
Multi-layer scaffold design (D = scaffold diameter; d = strand diameter; dis = strand distance).

In this work, a multi-layer 3D composite scaffold, based on a novel architectural design and established biomaterial formulations was developed as a strategy for the regeneration of hard periodontal tissue. The multi-layer cell-free scaffold was designed (Figure 1) and manufactured via a multi-material extrusion 3D printing. This approach offers the promising opportunity to fabricate complex tissue engineering scaffolds through the ability to co-print multiple biomaterials within the same construct by using independent dispensing systems [42]. Particularly, with respect to traditional fused deposition modeling, multi-extrusion 3D printing is a single-step approach, which is advantageous for the production of scaffolds able to mimic native tissue organization with high precision and reproducibility, starting from a computer aided design (CAD) model and thus allowing the development of patient-specific solutions [43,44].

Based on the outcomes of recent studies, the multi-layer scaffold was produced through a composite formulation, including a biodegradable polymeric matrix of PCL and a bioactive reinforcing phase of Sr-doped nano hydroxyapatite (Sr-nHA). The overall region-specific structure includes three compartments: a bottom one (C1) with high Sr-nHA content (20% *w*/*w*) coded as 20Sr-nHA to interface the alveolar bone tissue, an upper compartment (C3) with a lower ceramic content (10% *w*/*w*) coded as 10Sr-nHA to interface the cementum and an intermediate microporous one (C2) to guide the regeneration of the tissue between the alveolar bone and the cementum. Manufacturing process reproducibility, with respect to the original CAD model, as well as scaffold physicochemical and mechanical properties were assessed. Subsequently, in vitro biological performance, in terms of biocompatibility, alkaline phosphatase (ALP) activity and mineralization potential up to 28 days, was evaluated. In this regard, human bone osteosarcoma cells were used since they have been widely investigated for the initial in vitro appraisal of candidate biomaterials for bone tissue engineering applications [45,46,47].

## 2. Materials and Methods

### 2.1. Design and Manufacturing of the Multi-Layer Scaffold

The latest experts’ opinion evidenced that further optimization and refinement of the current periodontal biomaterial-based strategies are needed in order to functionally combine spatial, mechanical and biological benefits [44,48]. To support the still unmet demand for hybrid and multiphasic materials in dental practice, in this work a multi-layer composite scaffold intended to guide the regeneration of the hard periodontal tissue at the interface between the alveolar bone and the cementum was developed.

Cylindrical scaffolds with 7 mm diameter and 6 mm height were designed by using Solid EdgeTM 3D software, and then imported onto a dedicated platform (BioplotterRP 3.0 Software, EnvisionTEC, Gladbeck, Germany) in order to produce the slicing of the solid element. The entire architecture (see Table 1 for details) was made up of fourteen layers using a laydown pattern of 0/90° to create a porous structure. Each layer of the structure was printed continuously, following the geometry edge of the designed cylinder with a single extruded filament. Based on the recommendations and findings of recent studies [8,12,39], in order to increase pore interconnectivity (with pore dimensions between 150 and 500 μm) without compromising the mechanical properties, and allow a better in vitro performance, an offset distance equal to half the distance between strands was used (see Figure 1). Specifically, for the first 9 layers of the scaffold, intended to mimic the alveolar bone, the distance between two adjoining strands was set as 0.8 mm. In the C2 compartment of the scaffold, the distance between the strands was reduced from 0.8 to 0.7 mm, this in order to create a microporous structure intended to mimic the interface between the alveolar bone and the cementum; whereas the last five layers (C3) were produced using the same design of the first compartment and a lower ceramic content (10% *w*/*w*) within the PCL matrix. The same multi-layer architecture and inner pattern was followed to produce the bare PCL scaffold, which was used as a control.

Powdered poly-ε-caprolactone (PCL) with a relative molecular weight of 50 kDa and a particle size <600 μm was purchased from Polyscience Europe (Hirschberg, Germany). Sr-nHA, with strontium substitution at 20% molar weight, was produced as previously described [34] and supplied by the Biomaterials Innovation and Development Centre of Riga Technical University (Riga, Latvia).

The manufacturing of the scaffold was performed by using a 3D-Bioplotter system (EnvisionTEC, Gladbeck, Germany). Briefly, Sr-nHA was homogeneously mixed with the polymeric phase in two different concentrations (10:90 and 20:80 *w*/*w* respectively) using a mortar and pestle, then left overnight on a mechanical shaker (Stuart Scientific, Stone, UK), and ultimately 4 g of each of the powder mixtures were placed into stainless-steel cartridges (22G blunt tip needle, 0.4mm internal diameter) for processing.

Printing conditions (pressure, speed, pre- and post-flow, wait time) for the different formulations were then optimized and fixed as reported in Table 2.

### 2.2. Scaffold Physicochemical Characterization

In order to assess the chemical composition of the materials before and after the manufacturing process, the raw powders as well as the extruded filaments were analyzed through attenuated total reflectance Fourier transform infrared spectroscopy (ATR-FTIR). A Nicolet iS5 (Thermo Scientific, Stone, UK) equipped with an iD5 ATR diamond crystal window was used for the analyses. All the spectra were measured in the spectral range between 500 and 4000 cm^−1^. Thermogravimetric analysis (TGA) was performed in order to investigate the thermal characteristics as well as the composition of the printed composite materials. Particularly, TGA was useful to assess the actual Sr-nHA content within the polymeric matrix before and after the manufacturing process. A TGA2 METTLER TOLEDO™ with a resolution of 1 μg, weighing accuracy of 0.005% and weighing precision of 0.0025% was used to carry out the analyses. The extruded samples were tested in a range starting at room temperature (25 °C) and up to 800 °C, using a heating rate of 10 °C/min. Three samples of approximately 10 mg were tested for each formulation (PCL, PCL/10Sr-nHA and PCL/20Sr-nHA). The materials’ weight losses were measured and then normalized to the initial mass weight for each sample. The dedicated software STARe™ was used as an interface with the device to process the obtained information.

Moreover, the strands’ dimension and the top surface morphology of the 3D printed scaffolds were evaluated by a scanning electron microscope (SEM, Hitachi FE-SEM SU5000, Hitachi, Ltd., Tokyo, Japan). Prior to the image acquisition, the printed scaffolds were gold coated, using a manual sputter system (Quorum Technologies, Lewes, UK), and subsequently fixed on aluminum stubs. The coated samples were placed on the SEM stage and analyzed using a voltage of 7 kV and a working distance around 6 mm. All the images were collected through the equipment’s software. In addition, the topology and the internal architecture of the scaffolds were analyzed via microcomputed tomography (microCT). A Bruker Skyscan 1275 (Bruker, Kontich, Belgium), equipped with the Hamamatsu L11871 source and 3 MP active pixel CMOS (complementary metal oxide semiconductor) flat panel detector was used. The scanning was conducted under 30 kV and 250 µA in the microCT with an acquisition time of 49 ms and a pixel resolution of 10 µm. Reconstruction of the scans was performed using the NRecon software (Bruker, Kontich, Belgium), whereas qualitative visualization of the 3D architecture and the different phases of the composite material were performed and acquired through the CTvox software (Bruker, Kontich, Belgium).

3D scaffolds porosity (*P*) was calculated theoretically by using the following formula:

(1) $$P\;\left(\%\right)=1-\frac{\pi d_{1}^{2}}{4d_{2}d_{3}}\;\times \;100$$

in which, $d_{1}$ represents the diameter of the strand, $d_{2}$ the distance between strands and $d_{3}$ the height of the printed layer. Moreover, the porosity of the 3D printed scaffolds was calculated experimentally through the CTan software (Bruker, Kontich, Belgium), and where the actual dimensions of $d_{1}$, $d_{2}$ and $d_{3}$ were derived from the 2D sections of the microCT acquisitions. Subsequently, the software ImageJ was employed to obtain the measurements of the desired parameters, calculated as the average of three different samples.

In order to assess the mechanical performance of the multi-layer scaffolds, compression tests were carried out using an Instron 5500S testing machine (Instron, High Wycombe, UK), equipped with a 500 N load cell. The specimens (7 mm diameter and 6 mm height, *n* = 5) were initially subjected to a 2 N preload and then compressed across the longitudinal direction with a constant cross-head speed of 0.5 mm/min up to a deformation of 50% or until the safety range of the load cell, set at 490 N, was reached. The data collected were analyzed using Matlab and the Young’s modulus was calculated from the slope of the linear region of the stress–strain curve.

### 2.3. Scaffold Biological Characterization

#### 2.3.1. Cell Culture and Scaffold Seeding

Human bone osteosarcoma epithelial cells (U2OS Line, Thermo Fisher Scientific, UK) were cultured in McCoy’s growth medium (modified with l-glutamine, phenol-red and sodium bicarbonate) containing 10% of fetal bovine serum heat-inactivated (FBS-HI, Gibco) and 1% of the antibiotic agent (Penicillin-Streptomycin, Gibco). The medium was changed every second day. Cells at passages 7 were used for subsequent experiments. Before cell seeding, 3D scaffold compartments were sterilized following a two-step approach. Firstly, IPA (isopropyl alcohol, Honeywell, UK) was used to completely submerge the scaffolds for 15 min; after that, samples were washed three times with PBS (phosphate-buffered saline, Sigma Aldrich, Dorset, UK). In addition to this, 3D scaffolds were transferred into a 48 well plate and placed under a UV lamp (BioSan DNA/ RNA UV cleaner, λ = 253.7 nm) for 20 min. Samples were finally seeded by placing 5 μL of cell suspension (100,000 cells/mL) on their surface, and then incubated under standard conditions (37 °C, 5% CO_2_, 95% air, humidified environment); after 3 h 950 μL of fresh media was added into every well in order to reach a final volume of 1 mL.

#### 2.3.2. Cell Metabolic Activity, Morphology and Proliferation Test

MTT colorimetric assay was used to assess cell metabolic activity. After 1, 3 and 7 days post-seeding, 100 μL of the MTT solution (Sigma-Aldrich, 4% *w*/*v*), prepared following the supplier’s instructions, was added in each well (10% of total well volume) and samples (*n* = 5) were left in incubation for 3 h at 37 °C, 5% CO_2_ with protection from light exposure and in order to induce MTT reduction into purple formazan crystals. The solution was than replaced with 800 μL of IPA (Honeywell, UK) solvent and samples were left on a mechanical shaker at room temperature for 30 min. Subsequently, 200 μL of medium were dispensed into a 96 well plate and placed into a microplate reader (Sunrise™, Tecan, UK). The absorbance of each well was spectrophotometrically measured at a wavelength of 562 nm and measurements were taken in triplicate. Cell adhesion on the substrates was then evaluated after 3 and 7 days post-seeding.

Scaffold compartments were firstly washed with PBS; then, 1 mL of PFA (paraformaldehyde, Sigma-Aldrich, 4% in PBS, pH 7.4) was added into each well and left for 8 min at room temperature. Subsequently, each sample was washed three times with PBS before adding 800 μL of DAPI solution, used to completely cover the scaffolds’ volume. The DAPI working solution (300 nM) was prepared through a dilution of the DAPI (Thermo Fisher, Stone, UK) stock solution (5 mg/mL, 14.3 mM) in PBS. After 10 min of incubation in the dark and at room temperature, samples were washed twice with PBS and stored by adding 1–2 drops of the mounting medium (Sigma-Aldrich, Dorset, UK). An upright fluorescence microscope (Nikon ECLIPSE 80i) with a filter in the range between 350 and 450 nm was used for imaging. Different images were captured at two different magnifications using 20× and 10× objectives. In addition, the cells’ morphology was assessed via SEM analysis. After removing the culture media, samples were washed with sterile PBS and then chemically fixed with 2.5% glutaraldehyde (Sigma-Aldrich, Dorset, UK). Then, the fixing reagent was removed and the samples were further rinsed with PBS followed by post-fixation with 1% osmium tetroxide (Sigma-Aldrich, Dorset, UK) for 10 min. After rinsing with deionized water, the scaffolds were subsequently dehydrated using graded ethanol of 25%, 50%, 75%, 90% and 100%, then immersed into hexamethyldisilazane (HMDS, Sigma-Aldrich, Dorset, UK) and left for drying overnight before SEM investigation (20 A current and 5 kV voltage).

#### 2.3.3. Osteogenic Potential

To assess the scaffold osteogenic potential, alkaline phosphatase (ALP) activity (as an early osteogenic differentiation marker) was measured by using the ALP assay kit (Sigma-Aldrich, Dorset, UK) up to 28 days. After removing the media, samples were washed three times with PBS and fixed in 4% PFA. Subsequently, cells were washed with 0.1% PBS/Tween20^®^ solution and alkalinized with water/0.1M Tris solution (Sigma-Aldrich, Dorset, UK). After 25 min of incubation the alkaline solution was replaced with 1 mL of ALP solution (pNPP, Sigma-Aldrich, Dorset, UK) and samples were incubated for 30 min in the dark at room temperature. Of solution 100 µL was then taken from each well and placed in a clear-bottom 96-well plate to quantify the ALP activity. The reading was performed with a spectrometer (Tecan Scan™, Männedorf, Switzerland) at 405 nm. The results are based on the values obtained from a standard 8 points calibration curve, calculated by placing different known concentrations (from 200 to 0 ng/mL) of 0.1 M Tris/ALP into a 96-well plate. Additionally, ALP enzyme concentration was normalized over the total amount of proteins obtained through the BCA assay (ThermoFisher, Pierce™ BCA Protein Assay Kit, UK), in order to relate the measure to the effective number of cells in each sample. Following the procedure of the BCA kit, cells were washed three times with 1 mL of sterile PBS, then 1 mL of Tryple Express (Gibco TrypLE Thermofisher scientific, Scotland, UK)/1% Triton X-100 (Sigma Aldrich, St. Louis, MO, USA) was added and the plate was placed in incubation (37 °C) for 30 min. Afterwards, cell suspension was collected into sterile microtubes that were stored at −85 °C for 8 min. The suspensions were than microcentrifuged at 14,000 RFC at 4 °C for 10 min producing a separation between the solid cell bodies and the supernatant containing the released proteins. Aliquots of the supernatant of each sample (10 μL per well) were transferred into a 96 well plate and 200 μL of the BCA working reagent, previously prepared, were added as well. The plate was covered in foil and agitated for 5 min on a plate rotator (40 rpm) before being incubated for 30 min at 37 °C. The reading was performed with a spectrometer (Tecan Sunrise™, Theale, UK) at 562 nm. The results, obtained from a 9-points standard BCA curve, were calculated from different known concentrations between 2000 and 0 μg/mL. The dilutions were prepared from the BCA standard stock provided at a concentration of 2 μg/mL, whereas the diluent was the same used to dilute the proteins into cells suspension. Both tests (ALP/BCA assays) were performed in triplicate after 7, 14, 21 and 28 days.

#### 2.3.4. Calcium Detection

After removing the culture media, 3D scaffolds were fixed in 4% PFA and washed in PBS twice. Afterwards, samples were stained with 1 mL of Alizarin red solution (Sigma Aldrich, Dorset, UK), prepared in deionized water (2% *w*/*v*). Following an incubation time of 30 min at room temperature, the alizarin red solution was removed and scaffolds were washed multiple times and dried overnight at 60 °C in a 5% CO_2_ atmosphere. Samples were analyzed through optical microscopy using an Olympus™ CKX53 Inverted Microscope equipped with a 5× magnification objective. The experiment was performed at day 0, 7, 14 and 28.

### 2.4. Statistical Analysis

All the experiments were performed at least in triplicate. The results are reported as mean ± standard deviation (STD). Differences between groups were determined using a two-way ANOVA followed by a Dunnett’s test of significance, with a significance level of α = 5% (*p* values < 0.05).

## 3. Results and Discussion

### 3.1. Scaffold Composition, Design and Production

Single unit multi-layer composite scaffolds were produced in this work through a solvent free and single step manufacturing process. The composite scaffolds were developed by using a PCL polymeric matrix mainly for its good printability, biodegradability and for being an FDA approved biomaterial [49]. In relation to the ceramic phase, previous studies on Sr-containing biomaterials in various forms indicated that even small amounts of Sr (0.1 wt%) can enhance the osteoconductive properties of calcium phosphate, have a positive effect on bone mineralization, elevate the mechanical property of bone tissue and have crucial effects by inducing collagen type I synthesis [50,51,52,53]. Additionally, it has been reported that Sr can act via the calcium-sensing receptor and promote the transductions of the mitogen-activated protein kinase signaling. Thus, the substitution of calcium with strontium in the HA structure can lead to a ceramic material with improved bioactivity and osteogenic properties [32,54]. Furthermore, based on the promising findings of a recent study, according to which the use of Sr-nHA enhanced the osteogenic potential and mineralization of human mesenchymal stromal cells, in this work Sr-nHA (2.46 wt%) was selected as reinforcing phase for the PCL polymeric matrix [34]. The content of the nanosized ceramic phase was chosen to decrease along the structure going from the bottom (alveolar bone, 20% *w*/*w*) to the top (cementum, 10% *w*/*w*).

In relation to the scaffold design, a novel architecture was developed to mimic the multi-phasic architecture of hard periodontal tissue. After an initial optimization of the printing process, multi-layer scaffolds were manufactured according to the parameters reported in Table 2.

No major differences were recorded in terms of printing temperature between PCL and PCL/10Sr-nHA, whereas the increased content of Sr-nHA powder (above 10 wt%) required further optimization of the printing conditions. Particularly, higher printing temperature, but still below the degradation temperature of the PCL matrix, and pressure were needed to guarantee a consistent and defect-free extrusion process of the composite formulation. As reported by previous studies, this behavior might be due to the polymer acting as nucleation site for the ceramic phase, and thus requiring different processing conditions [55,56].

### 3.2. Characterization of Multi-Layered Scaffolds

**Figure 2:**
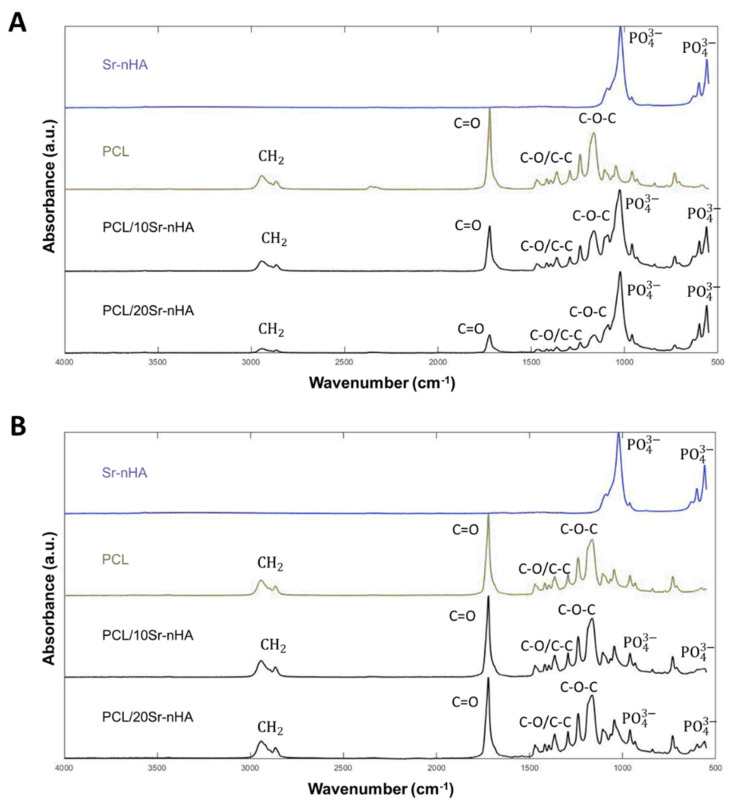
FTIR spectra of (**A**) raw powders and (**B**) extruded filaments deriving from the additive manufacturing process (spectra of Sr-nHA powder is included for comparison purpose).

ATR-FTIR was performed in order to assess the chemical composition, in terms of functional groups, of both precursors in the form of powders and extruded materials, thus before and after the manufacturing process. In Figure 2A PCL spectra shows the characteristic bands of this polymer. Specifically, the C–H stretching (2943 and 2865 cm^−1^), the C=O carbonyl group at 1720 cm^−1^, and the CH_2_ deformation band at 1165–1468 cm^−1^, the backbone C–O and C–C stretching in the crystalline phase at 1293 cm^−1^ and the C–O–C symmetric and asymmetric band at 1239 cm^−1^, 1164 cm^−1^, 1107 cm^−1^ and 1047 cm^−1^ [57]. In relation to the composite powders (PCL/10Sr-nHA and PCL/20Sr-nHA), the Sr-nHA hexagonal crystalline structure produced intensive IR absorption bands at 560 and 600 cm^−1^ and at 1041 cm^−1^, deriving from the O–H and $PO_{4}^{3-}$ groups [34,35].

As is clearly visible in Figure 2B, the presence of the polymeric as well as the ceramic phase was still evident in the extruded composite formulations, suggesting that the 3D manufacturing process did not affect the chemical composition of the printed materials, and proving the efficacy of the solvent free mechanical mixing.

**Figure 3:**
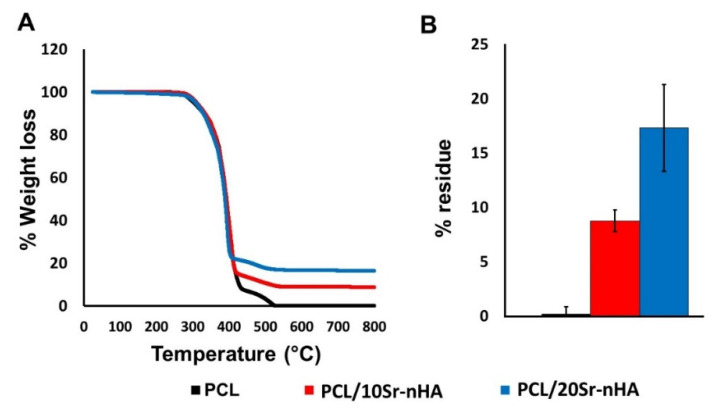
(**A**) TGA curves for the 3D printed filaments and (**B**) resulting ceramic residues (%) after the extrusion process.

The extruded materials were then analyzed through TGA analysis (see Figure 3). PCL shows a complete degradation with a reaction that started at around 300 °C and ended at 550 °C, with a maximum decomposition peak at 490.2 °C. Both composite samples displayed a similar weight loss profile, evidencing that the Sr-nHA presence did not affect the composite materials’ thermal stability [58]. Moreover, the residue of ceramic content for PCL/10Sr-nHA and PCL/20Sr-nHA was 8.75 ± 0.94 wt% and 17.3 ± 3.45 wt% respectively, proving to be within the wt% of the inorganic component of the as designed formulations, and further demonstrating how the hot melt extrusion approach did not affect the biomaterials’ thermal behavior.

**Figure 4:**
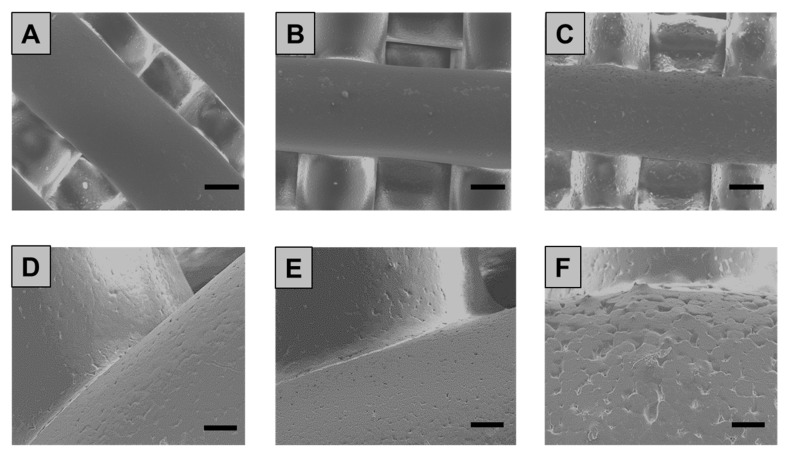
SEM images of the 3D printed PCL (**A**,**D**), PCL/10Sr-nHA (**B**,**E**) and PCL/Sr20-nHA (**C**,**F**) scaffolds ((**A**–**C**) scale bar = 200 µm and (**D**–**F**) scale bar = 50 µm).

The morphology and topology of the 3D printed scaffolds were then assessed both qualitatively and quantitatively. According to the SEM analysis, printed scaffolds (see Figure 4) showed well-aligned and parallel filaments, both in the horizontal and perpendicular planes, with an average strand diameter equal to 461 ± 10 µm, 455 ± 7 µm and 433 ± 13 µm for PCL, PCL/10Sr-nHA and PCL/20Sr-nHA respectively. These values coherently matched the theoretical parameters of the CAD design, indicating the high reproducibility of the manufacturing process. Additionally, from the high magnification SEM images (see Figure 4D–F), it is possible to observe how the presence of the ceramic phase affects the strand surface at the microscopic level. In accordance to the findings reported by Bruyas et al. and Huang et al., by increasing the ceramic content the composite filaments’ surface appeared less smooth than the bare PCL and with more pronounced micropores, whose formation might be due to the collision of ceramic particles with PCL crystalline regions growing during solidification [58,59].

**Figure 5:**
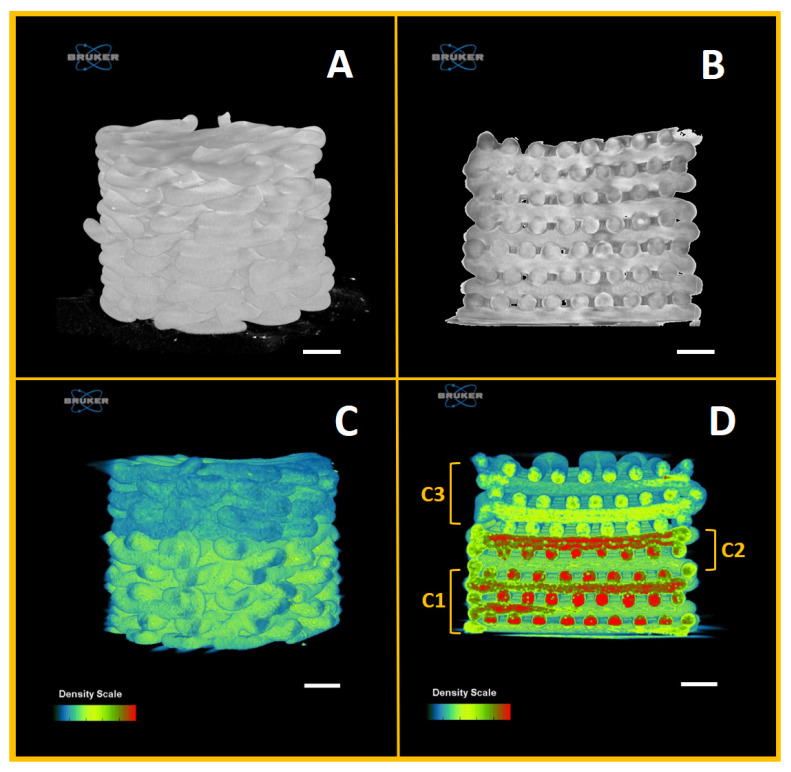
microCT reconstructions of: (**A**) PCL scaffold, (**B**) PCL scaffold cross section, (**C**) multi-layer PCL/Sr-nHA scaffold and (**D**) multi-layer PCL/Sr-nHA scaffold cross section where the C1, C2 and C3 compartments are indicated.

In addition, a non-destructive characterization technique was used to investigate the 3D printed multi-layer scaffold architecture as well as the inner geometry. According to the microCT reconstructions (Figure 5), the optimized printing conditions led to the production of 3D scaffolds with continuous filaments, deposited in a layer-by-layer fashion, along with an interconnected structure within each layer (see Figure 5A–C). Towards the development of tissue-like scaffolds, interconnectivity has been demonstrated to be an essential requirement, crucial to aid cell growth, migration and proliferation [60,61]. Furthermore, according to the 3D reconstructions of the cross section, the multi-layer scaffold showed the homogeneous distribution of the ceramic phase within the polymeric matrix. Additionally, it can be observed (see Figure 5D) how individual layers had a distinct architecture seamlessly integrated. Particularly, the C2 layers resulted in being strongly connected with the upper C3 and bottom C1 layers. This is of great importance to ensure the mechanical integration at the junctions and prevent any possibility of delamination after implantation [62,63], yet allowing the cross-talk within the overall microenvironment.

Regarding the printed scaffolds’ porosity, whose data are reported in Table 3, it can be observed that the experimental porosity values, derived from the microCT analysis, for both PCL and multi-layer PCL/Sr-nHA scaffolds were consistent with those calculated using the theoretical formula and within the range of dentoalveolar bone [12]. Additionally, similarly to the finding reported by Bittner et al., no differences were observed following the incorporation of the ceramic phase on the scaffold total porosity in comparison to the pure PCL samples [42].

**Figure 6:**
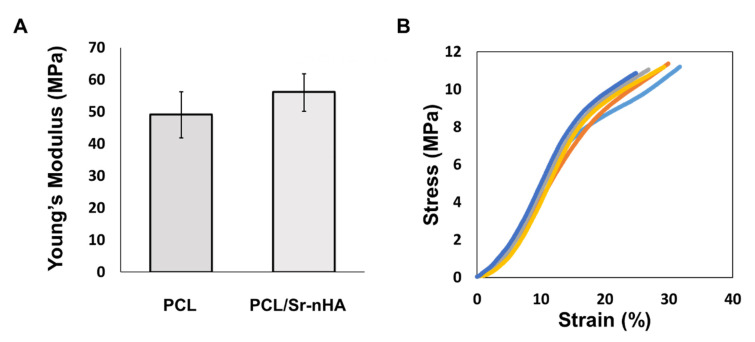
(**A**) Young’s modulus values for PCL and PCL/Sr-nHA multi-layer scaffolds and (**B**) representative stress/strain curves (*n* = 5) for PCL/Sr-nHA multi-layered scaffolds tested in compression.

Considering prior literature, it has been demonstrated that the mechanical properties of human bone depend greatly on patient age, activity and location in the body. As result, the compressive modulus values have been found to vary in a range rather being ascribed to a single value [64]. In the case of low load-bearing applications like alveolar bone, the scaffold stability and the appropriate 3D shape for functional and aesthetic reason have been considered more important than high mechanical properties [65]. The multi-layer PCL/Sr-nHA scaffolds produced in this work were then characterized via uniaxial compression tests. The composite samples displayed values of Young’s modulus comparable with those of pure PCL (Figure 6A), indicating that the inclusion of the ceramic phase up to 20% (*w*/*w*) broadly affected the scaffold elastic modulus in compression. As demonstrated by the findings reported in other studies, this outcome might be the consequence of the mechanical mixing rather than chemical blending of the two phases, in which the polymer act as a matrix, surrounding the ceramic nanoparticles [34,66,67]. However, the poor adhesion at the interface between different compartments has been one of the main issues of previous multi-layer approaches, and where the combination of different materials within the same structure was considered. As can be observed from Figure 6B, different multi-layer samples displayed stress–strain curves with a very similar trend. This result can be considered a clear indication of the scaffolds ability to maintain structural integrity under compressive loadings, and hence indicating the suitability of the manufacturing approach to fabricate 3D scaffolds with reproducible mechanical performance.

### 3.3. In Vitro Biological Performance

**Figure 7:**
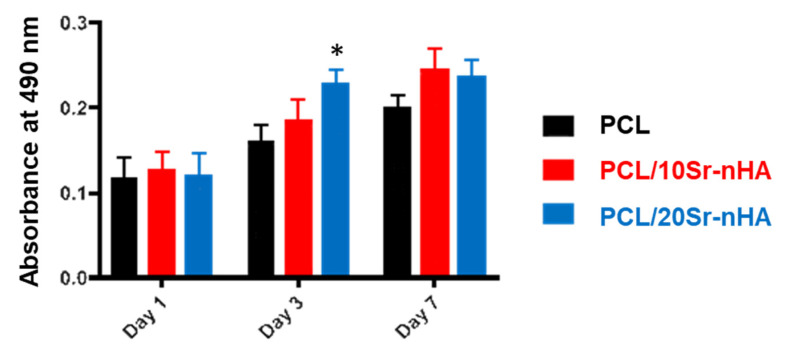
Absorbance values of the MTT assay evaluated up to 7 days for PCL, PCL/10Sr-nHA and PCL/20Sr-nHA scaffolds in culture with U2OS cells (data are presented as mean ± standard deviation; *n* = 3, *p* < 0.05 (*)).

The biological performance of the different material formulations within the multi-layer 3D scaffold was then assessed in vitro by using the human osteosarcoma U2OS cell line. Viability, attachment, osteogenic and mineralization potential were evaluated. As shown by the MTT colorimetric assay results, reported in Figure 7, the absorbance values increased with time, evidencing a progressive growth of the U2OS cells’ metabolic activity after 7 days in culture. By comparing the composite samples tested, no significant differences were recorded within the groups and with respect to PCL-based scaffolds at both day 1 and 7. Only at day 3, the metabolic activity of the specimens with greater Sr-nHA content was significantly higher with respect to the other two. However, being PCL and FDA approved biomaterial it can be stated that both scaffold compositions proposed in our study sustain U2OS cell proliferation.

**Figure 8:**
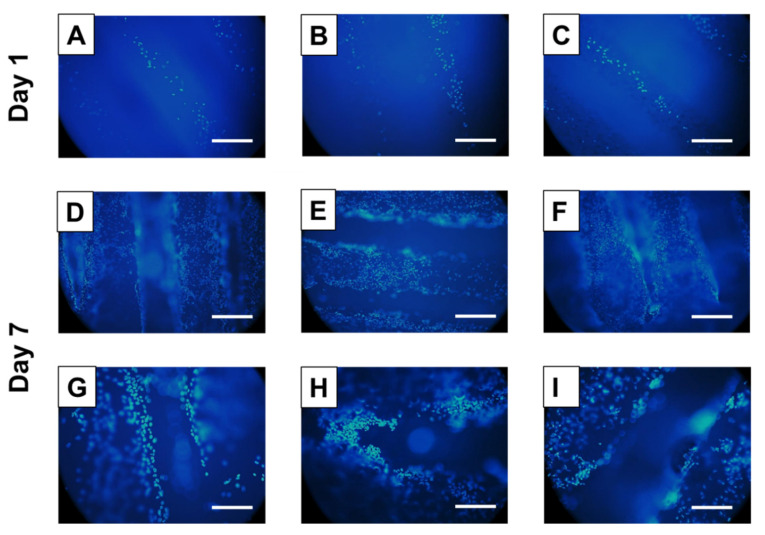
DAPI staining of U2OS cells seeded on PCL (**A**,**D**,**G**), PCL/10Sr-nHA (**B**,**E**,**H**) and PCL/20Sr-nHA (**C**,**F**,**I**) after 1 and 7 days in culture; ((**A**–**F**) scale bar = 400 µm; (**G**–**I**) scale bar = 200 µm).

These results were further confirmed by a DAPI cytofluorometric assay (see Figure 8). U2OS proliferation on the 3D additively manufactured scaffolds was investigated for 7 days. Despite the PCL autofluorescence in the same range of the DAPI reactivity, this assay enabled U2OS nuclei visualization and adhesion on both scaffolds’ surface and within the inner architecture [68,69].

U2OS proliferated considerably well on all the scaffolds over time, with a higher cell density on the composite-based scaffold surface after 7 days in culture. Additionally, it has been observed that cells were slightly more concentrated in the intersection area between two orthogonal strands on all sample types. This result suggests that, in accordance to previous studies, the shifted design pattern as well as its interconnected porosity allowed U2OS migration into the sample’s depth, leading to cell growth into the internal 3D structure [14,70].

**Figure 9:**
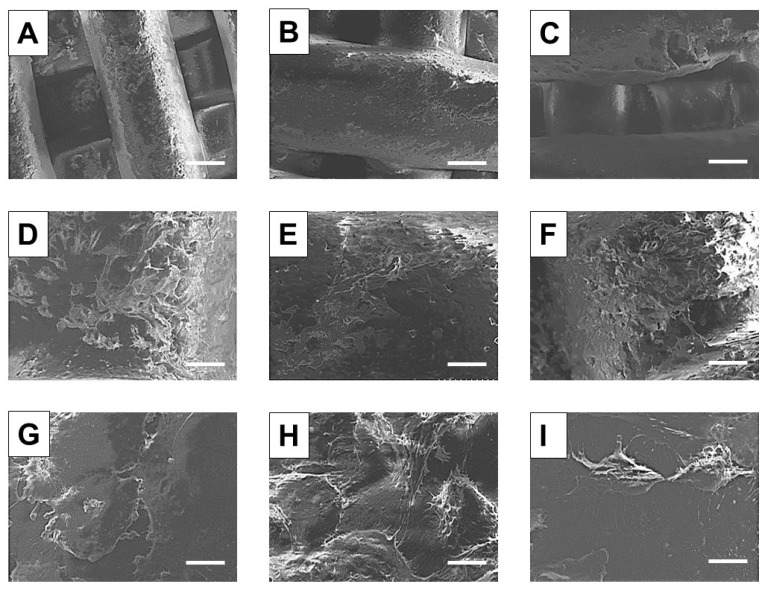
SEM micrographs of U2OS cells seeded on PCL (**A**,**D**,**G**), PCL/10Sr-nHA (**B**,**E**,**H**) and PCL/20Sr-nHA (**C**,**F**,**I**) after 14 days in culture; ((**A**–**C**) scale bar = 200 µm; (**D**–**F**) scale bar = 40 µm; (**G**–**I**) scale bar = 8 µm).

In order to assess the cell–material interaction, morphological investigation via SEM analysis was conducted on the additively manufactured samples 14 days after seeding. From the low magnification images (Figure 9A–F)) cell distribution and attachment can be observed. Cell growth was oriented across the strand direction, and with a surface coverage that resulted in being more pronounced on the sample with higher ceramic content (Figure 9C–F) in comparison to the control (Figure 9A–D). Moreover, as shown in the higher magnification images, cell–cell contact and filopodia formation was established particularly on the composite-based samples (Figure 9H,I), whereas on the PCL scaffold surface cells displayed a more rounded shape and the filopodia were almost indiscernible. This outcome was mainly ascribed to the scaffold composition, since it is widely reported as the essential role of nHA and Sr-containing nHA to promote attachment, proliferation and osteoblastic differentiation [34,50,53]. Furthermore, the actual roughness of the as printed composite scaffolds’ (see Figure 4E–F) could be identified as another reason determining the positive cell–substrate interaction informed in Figure 9 [71,72,73].

**Figure 10:**
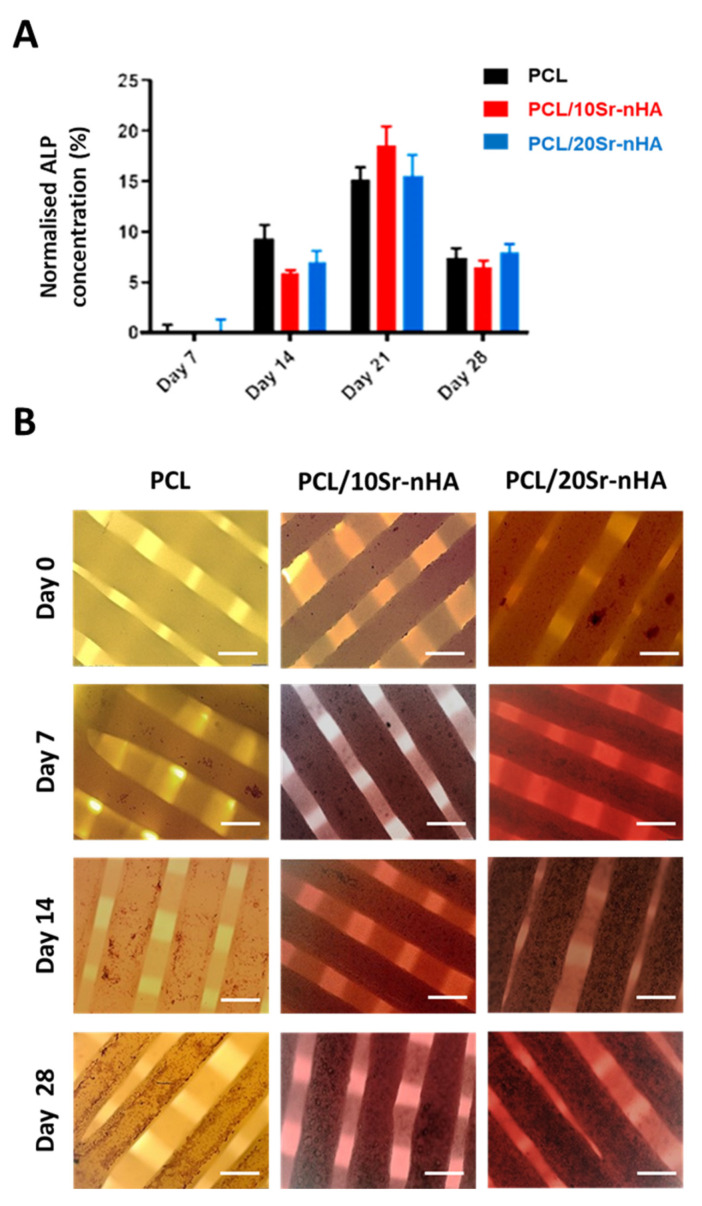
(**A**) Normalized ALP activity of the PCL, PCL/10Sr-nHA and PCL/20Sr-nHA samples up to 28 days in culture with U2OS cells (data are presented as mean ± standard deviation. *n* = 6) and (**B**) optical microscopy images of cell-seeded scaffolds showing calcified nodules stained with Alizarin red after 0, 7, 14 and 28 days (scale bar = 400 µm).

Regarding the scaffold osteogenic potential, the ALP assay was performed up to 28 days. ALP is an early marker of osteogenic differentiation and its expression decreases as bone tissue matures [74]. In Figure 10A the concentration of ALP normalized on the total protein content is shown. ALP activity in all groups increased up to day 21, and decreased thereafter. However, and similarly to recent studies, in the present work the inclusion of Sr-nHA in the polymeric matrix was not observed to sustain the ALP activity in comparison to the control, and at each time point. With respect to the findings reported by Zhou et al., where the expression of ALP increased for 2 weeks and then decreased, in our study ALP expression increased up to 3 weeks before decreasing [75]. This outcome could be related to the type of cells used. In fact, according to Wilkesmann et al. U2OS cells have a significantly slow osteogenic activity in comparison to primary human osteoblasts and primary mesenchymal stromal cells, indicating the strong influence that cell types have on ALP expression [45].

Mineralization potential was then assessed qualitatively by Alizarin red assay. Alizarin red, an anthraquinone dye, has significant binding affinity to deposited calcium. Hence, it is normally used to verify the presence of matrix mineralization, and as such, it is considered an early stage marker for osteogenesis [76]. In our study, Alizarin red assay enabled us to prove the extent of mineralization activity supported by 3D printed scaffolds. The stain color in all groups became darker by increasing time intervals, suggesting that more calcium was deposited onto the scaffolds over time (Figure 10B). There was a detectable difference in both the red staining color and presence of inorganic calcium components over the surface of composite-based samples; the inorganic deposits resulted in being bigger in size and more uniformly distributed in comparison to the control. In agreement with recent literature, this observation demonstrates that greater mineralization was generated by the Sr-containing scaffolds with respect to the monophasic PCL, indicating both PCL/10Sr-nHA and PCL/20Sr-nHA samples as a promising platform for U2OS growth and osteogenesis [34,53,77].

## 4. Conclusions

To repair periodontal defects, advanced methodologies are required to manufacture scaffolds able to replicate the multi-layer architecture of the native tissue. In this study, we applied an emergent manufacturing technology able to produce a multi-layer composite scaffold varying simultaneously the composition and the geometry of the three former compartments, thus better mimicking the key features of the native hard periodontal tissue. The single step procedure allowed the achievement of excellent integrity among the different compartments guaranteeing mechanical stability of the whole structure. Overall, the results indicate that the additively manufactured scaffolds incorporated porosity levels and compressive properties within the range of dentoalveolar bone tissue. Furthermore, in vitro cell tests revealed a high U2OS cells viability and confirmed the important role of the inorganic phase (Sr-nHA) towards the mineralization process.

In conclusion, the manufacturing process used revealed a highly versatile method to create 3D scaffolds with reproducible mechanical properties, and favorable biological performance, avoiding drawbacks associated to poor integration of layers and cytotoxicity associated to solvent residuals. Moving forward, the manufacturing strategy applied in this study may serve as a platform for the development of more complex and patient-specific substitutes, allowing the inclusion of additional biomaterials and bioactive cues that better match the overall architecture of the native periodontal tissue, including the gingiva, and opening a new horizon in the tissue engineering field, beyond dental applications.

## Figures and Tables

**Table 1:** 3D scaffold composition, design parameters and architecture.

Scaffold Code	Architecture	Strand Distance	Material Composition
PCL	C3: layer 10 to 14	0.8 mm	PCL
	C2: layer 7 to 9	0.7 mm	
PCL/Sr-nHA			PCL/10Sr-nHA (10% *w*/*w*)
	C1: layer 1 to 6	0.8 mm	PCL/20Sr-nHA (20% *w*/*w*)
			PCL/20Sr-nHA (20% *w*/*w*)

**Table 2:** 3D printing parameters.

	Temperature (°C)	Pressure (bar)	Speed (mm/s)	Pre-Flow (s)	Post-Flow (s)	Wait Time (s)
PCL	130	5.3	0.7	0.45	0.1	10
PCL/10Sr-nHA	130	6.3	0.5	0.75	0.2	10
PCL/20Sr-nHA	140	6.5	0.4	0.75	0.2	10

**Table 3:** Theoretical and experimental porosity values of the overall 3D scaffolds (PCL and PCL/Sr-nHA) and for each compartment (C1, C2 and C3) within the overall structure.

CODE	Total Porosity (%)—Overall		Total Porosity (%)—Compartments	
	Theoretical	Experimental	Theoretical	Experimental
**PCL**	C3: 41.56 C2: 32.50 C1: 41.56	37.36 ± 0.99	C3: 41.56	C3: 40.2 ± 1.5
				C2: 30.3 ± 1.2
			C2: 32.50	C1: 36.6 ± 1.3
**PCL/Sr-nHA**		39.02 ± 0.51		C3: 43.6 ± 2.5
			C1: 41.56	C2: 31.6 ± 1.7
				C1: 42.5 ± 0.9

## References

[1] Peres M.A., Macpherson L.M.D., Weyant R.J., Daly B., Venturelli R., Mathur M.R., Listl S., Celeste R.K., Guarnizo-Herreño C.C., Kearns C. (2019). Oral diseases: A global public health challenge. Lancet.

[2] Ito R., Kubota K., Inui A., Nakagawa H., Kon T., Narita N., Tamura Y., Oyama T., Satake A., Furudate K. (2017). Oral-maxillofacial trauma of a geriatric population in a super-ageing country. Dent. Traumatol..

[3] Thomson W.M., Ma S. (2014). An ageing population poses dental challenges. Singap. Dent. J..

[4] Razak P.A., Richard K.M.J., Thankachan R.P., Hafiz K.A.A., Kumar K.N., Sameer K.M. (2014). Geriatric Oral Health: A Review Article. J. Int. Oral Health.

[5] Frencken J.E., Sharma P., Stenhouse L., Green D., Laverty D., Dietrich T. (2017). Global epidemiology of dental caries and severe periodontitis—A comprehensive review. J. Clin. Periodontol..

[6] Herford A.S., Nguyen K. (2015). Complex bone augmentation in alveolar ridge defects. Oral Maxillofac. Surg. Clin. N. Am..

[7] Lee H.-S., Byun S.-H., Cho S.-W., Yang B.-E. (2019). Past, Present, and Future of Regeneration Therapy in Oral and Periodontal Tissue: A Review. Appl. Sci..

[8] Liang Y., Luan X., Liu X. (2020). Recent advances in periodontal regeneration: A biomaterial perspective. Bioact. Mater..

[9] Titsinides S., Agrogiannis G., Karatzas T. (2019). Bone grafting materials in dentoalveolar reconstruction: A comprehensive review. Jpn. Dent. Sci. Rev..

[10] Henkel J., Woodruff M.A., Epari D.R., Steck R., Glatt V., Dickinson I.C., Choong P.F.M., Schuetz M.A., Hutmacher D.W. (2013). Bone Regeneration Based on Tissue Engineering Conceptions—A 21st Century Perspective. Bone Res..

[11] Shimauchi H., Nemoto E., Ishihata H., Shimomura M. (2013). Possible functional scaffolds for periodontal regeneration. Jpn. Dent. Sci. Rev..

[12] Asa’ad F., Pagni G., Pilipchuk S.P., Giannì A.B., Giannobile W.V., Rasperini G. 3D-Printed Scaffolds and Biomaterials: Review of Alveolar Bone Augmentation and Periodontal Regeneration Applications. https://www.hindawi.com/journals/ijd/2016/1239842/.

[13] Iviglia G., Kargozar S., Baino F. (2019). Biomaterials, Current Strategies, and Novel Nano-Technological Approaches for Periodontal Regeneration. J. Funct. Biomater..

[14] Yilgor P., Sousa R.A., Reis R.L., Hasirci N., Hasirci V. (2008). 3D Plotted PCL Scaffolds for Stem Cell Based Bone Tissue Engineering. Macromol. Symp..

[15] Dwivedi R., Kumar S., Pandey R., Mahajan A., Nandana D., Katti D.S., Mehrotra D. (2020). Polycaprolactone as biomaterial for bone scaffolds: Review of literature. J. Oral Biol. Craniofacial Res..

[16] Chen Q., Zhu C., Thouas G.A. (2012). Progress and challenges in biomaterials used for bone tissue engineering: Bioactive glasses and elastomeric composites. Prog. Biomater..

[17] Bayani M., Torabi S., Shahnaz A., Pourali M. (2017). Main properties of nanocrystalline hydroxyapatite as a bone graft material in treatment of periodontal defects. A review of literature. Biotechnol. Biotechnol. Equip..

[18] Brunello G., Panda S., Schiavon L., Sivolella S., Biasetto L., Del Fabbro M. (2020). The Impact of Bioceramic Scaffolds on Bone Regeneration in Preclinical In Vivo Studies: A Systematic Review. Materials.

[19] Salinas A.J., Esbrit P., Vallet-Regí M. (2012). A tissue engineering approach based on the use of bioceramics for bone repair. Biomater. Sci..

[20] Mancuso E., Bretcanu O.A., Marshall M., Birch M.A., McCaskie A.W., Dalgarno K.W. (2017). Novel bioglasses for bone tissue repair and regeneration: Effect of glass design on sintering ability, ion release and biocompatibility. Mater. Des..

[21] Rotbaum Y., Puiu C., Rittel D., Domingos M. (2019). Quasi-static and dynamic in vitro mechanical response of 3D printed scaffolds with tailored pore size and architectures. Mater. Sci. Eng. C.

[22] Kashirina A., Yao Y., Liu Y., Leng J. (2019). Biopolymers as bone substitutes: A review. Biomater. Sci..

[23] Ginebra M.-P., Espanol M., Maazouz Y., Bergez V., Pastorino D. (2018). Bioceramics and bone healing. Effort Open Rev..

[24] Zhuang Y., Lin K., Yu H. (2019). Advance of Nano-Composite Electrospun Fibers in Periodontal Regeneration. Front. Chem..

[25] Zhu L., Luo D., Liu Y. (2020). Effect of the nano/microscale structure of biomaterial scaffolds on bone regeneration. Int. J. Oral Sci..

[26] Tcacencu I., Rodrigues N., Alharbi N., Benning M., Toumpaniari S., Mancuso E., Marshall M., Bretcanu O., Birch M., McCaskie A. (2018). Osseointegration of porous apatite-wollastonite and poly(lactic acid) composite structures created using 3D printing techniques. Mater. Sci. Eng. C.

[27] Bottino M.C., Thomas V., Janowski G.M. (2011). A novel spatially designed and functionally graded electrospun membrane for periodontal regeneration. Acta Biomater..

[28] Kasaj A., Willershausen B., Reichert C., Röhrig B., Smeets R., Schmidt M. (2008). Ability of nanocrystalline hydroxyapatite paste to promote human periodontal ligament cell proliferation. J. Oral Sci..

[29] Higuchi J., Fortunato G., Woźniak B., Chodara A., Domaschke S., Męczyńska-Wielgosz S., Kruszewski M., Dommann A., Łojkowski W. (2019). Polymer Membranes Sonocoated and Electrosprayed with Nano-Hydroxyapatite for Periodontal Tissues Regeneration. Nanomaterials.

[30] Sattary M., Khorasani M.T., Rafienia M., Rozve H.S. (2018). Incorporation of nanohydroxyapatite and vitamin D3 into electrospun PCL/Gelatin scaffolds: The influence on the physical and chemical properties and cell behavior for bone tissue engineering. Polym. Adv. Technol..

[31] Kanaya S., Nemoto E., Sakisaka Y., Shimauchi H. (2013). Calcium-mediated increased expression of fibroblast growth factor-2 acts through NF-κB and PGE2/EP4 receptor signaling pathways in cementoblasts. Bone.

[32] Zhang S., Dong Y., Chen M., Xu Y., Ping J., Chen W., Liang W. (2020). Recent developments in strontium-based biocomposites for bone regeneration. J. Artif. Organs.

[33] Ni G.-X., Shu B., Huang G., Lu W.W., Pan H.-B. (2012). The effect of strontium incorporation into hydroxyapatites on their physical and biological properties. J. Biomed. Mater. Res. Part B Appl. Biomater..

[34] Pierantozzi D., Scalzone A., Jindal S., Stīpniece L., Šalma-Ancāne K., Dalgarno K., Gentile P., Mancuso E. (2020). 3D printed Sr-containing composite scaffolds: Effect of structural design and material formulation towards new strategies for bone tissue engineering. Compos. Sci. Technol..

[35] Tsai S.-W., Yu W.-X., Hwang P.-A., Hsu Y.-W., Hsu F.-Y. (2019). Fabrication and Characteristics of PCL Membranes Containing Strontium-Substituted Hydroxyapatite Nanofibers for Guided Bone Regeneration. Polymers.

[36] Liu J., Ruan J., Weir M.D., Ren K., Schneider A., Wang P., Oates T.W., Chang X., Xu H.H.K. (2019). Periodontal Bone-Ligament-Cementum Regeneration via Scaffolds and Stem Cells. Cells.

[37] Ivanovski S., Vaquette C., Gronthos S., Hutmacher D.W., Bartold P.M. (2014). Multiphasic Scaffolds for Periodontal Tissue Engineering. J. Dent Res..

[38] Goudouri O.-M., Kontonasaki E., Boccaccini A.R., Tayebi L., Moharamzadeh K. (2017). 17–Layered Scaffolds for Periodontal Regeneration. Biomaterials for Oral and Dental Tissue Engineering.

[39] Rasperini G., Pilipchuk S.P., Flanagan C.L., Park C.H., Pagni G., Hollister S.J., Giannobile W.V. (2015). 3D-printed Bioresorbable Scaffold for Periodontal Repair. J. Dent Res..

[40] Vikram Singh A., Hasan Dad Ansari M., Wang S., Laux P., Luch A., Kumar A., Patil R., Nussberger S. (2019). The Adoption of Three-Dimensional Additive Manufacturing from Biomedical Material Design to 3D Organ Printing. Appl. Sci..

[41] van Kampen K.A., Scheuring R.G., Terpstra M.L., Levato R., Groll J., Malda J., Mota C., Moroni L., Reis R.L. (2019). Biofabrication: From Additive Manufacturing to Bioprinting. Encyclopedia of Tissue Engineering and Regenerative Medicine.

[42] Bittner S.M., Smith B.T., Diaz-Gomez L., Hudgins C.D., Melchiorri A.J., Scott D.W., Fisher J.P., Mikos A.G. (2019). Fabrication and mechanical characterization of 3D printed vertical uniform and gradient scaffolds for bone and osteochondral tissue engineering. Acta Biomater..

[43] Rider P., Kačarević Ž.P., Alkildani S., Retnasingh S., Schnettler R., Barbeck M. (2018). Additive Manufacturing for Guided Bone Regeneration: A Perspective for Alveolar Ridge Augmentation. Int. J. Mol. Sci..

[44] Carter S.-S.D., Costa P.F., Vaquette C., Ivanovski S., Hutmacher D.W., Malda J. (2017). Additive Biomanufacturing: An Advanced Approach for Periodontal Tissue Regeneration. Ann. Biomed. Eng..

[45] Wilkesmann S., Fellenberg J., Nawaz Q., Reible B., Moghaddam A., Boccaccini A.R., Westhauser F. (2020). Primary osteoblasts, osteoblast precursor cells or osteoblast-like cell lines: Which human cell types are (most) suitable for characterizing 45S5-bioactive glass?. J. Biomed. Mater. Res. Part A.

[46] Bouet G., Marchat D., Cruel M., Malaval L., Vico L. (2015). In vitro three-dimensional bone tissue models: From cells to controlled and dynamic environment. Tissue Eng. Part B Rev..

[47] Roseti L., Parisi V., Petretta M., Cavallo C., Desando G., Bartolotti I., Grigolo B. (2017). Scaffolds for Bone Tissue Engineering: State of the art and new perspectives. Mater. Sci. Eng. C Mater. Biol. Appl..

[48] Vaquette C., Pilipchuk S.P., Bartold P.M., Hutmacher D.W., Giannobile W.V., Ivanovski S. (2018). Tissue Engineered Constructs for Periodontal Regeneration: Current Status and Future Perspectives. Adv. Healthc. Mater..

[49] Kundu J., Pati F., Hun Jeong Y., Cho D.-W. (2013). Biomaterials for Biofabrication of 3D Tissue Scaffolds. Biofabrication.

[50] Panzavolta S., Torricelli P., Casolari S., Parrilli A., Fini M., Bigi A. (2018). Strontium-Substituted Hydroxyapatite-Gelatin Biomimetic Scaffolds Modulate Bone Cell Response. Macromol. Biosci..

[51] Saidak Z., Marie P.J. (2012). Strontium signaling: Molecular mechanisms and therapeutic implications in osteoporosis. Pharmacol. Ther..

[52] Zhang W., Shen Y., Pan H., Lin K., Liu X., Darvell B.W., Lu W.W., Chang J., Deng L., Wang D. (2011). Effects of strontium in modified biomaterials. Acta Biomater..

[53] Liu D., Nie W., Li D., Wang W., Zheng L., Zhang J., Zhang J., Peng C., Mo X., He C. (2019). 3D printed PCL/SrHA scaffold for enhanced bone regeneration. Chem. Eng. J..

[54] Marx D., Rahimnejad Yazdi A., Papini M., Towler M. (2020). A review of the latest insights into the mechanism of action of strontium in bone. Bone Rep..

[55] Jiao Z., Luo B., Xiang S., Ma H., Yu Y., Yang W. (2019). 3D printing of HA / PCL composite tissue engineering scaffolds. Adv. Ind. Eng. Polym. Res..

[56] Navarro-Baena I., Sessini V., Dominici F., Torre L., Kenny J.M., Peponi L. (2016). Design of biodegradable blends based on PLA and PCL: From morphological, thermal and mechanical studies to shape memory behavior. Polym. Degrad. Stab..

[57] Neufurth M., Wang X., Wang S., Steffen R., Ackermann M., Haep N.D., Schröder H.C., Müller W.E.G. (2017). 3D printing of hybrid biomaterials for bone tissue engineering: Calcium-polyphosphate microparticles encapsulated by polycaprolactone. Acta Biomater..

[58] Huang B., Caetano G., Vyas C., Blaker J., Diver C., Bártolo P. (2018). Polymer-Ceramic Composite Scaffolds: The Effect of Hydroxyapatite and β-tri-Calcium Phosphate. Materials.

[59] Bruyas A., Lou F., Stahl A.M., Gardner M., Maloney W., Goodman S., Yang Y.P. (2018). Systematic characterization of 3D-printed PCL/β-TCP scaffolds for biomedical devices and bone tissue engineering: Influence of composition and porosity. J. Mater. Res..

[60] Sobral J.M., Caridade S.G., Sousa R.A., Mano J.F., Reis R.L. (2011). Three-dimensional plotted scaffolds with controlled pore size gradients: Effect of scaffold geometry on mechanical performance and cell seeding efficiency. Acta Biomater..

[61] Bružauskaitė I., Bironaitė D., Bagdonas E., Bernotienė E. (2016). Scaffolds and cells for tissue regeneration: Different scaffold pore sizes—different cell effects. Cytotechnology.

[62] Nooeaid P., Salih V., Beier J.P., Boccaccini A.R. (2012). Osteochondral tissue engineering: Scaffolds, stem cells and applications. J. Cell. Mol. Med..

[63] Levingstone T.J., Matsiko A., Dickson G.R., O’Brien F.J., Gleeson J.P. (2014). A biomimetic multi-layered collagen-based scaffold for osteochondral repair. Acta Biomater..

[64] Morgan E.F., Unnikrisnan G.U., Hussein A.I. (2018). Bone Mechanical Properties in Healthy and Diseased States. Annu. Rev. Biomed. Eng..

[65] Matsuno T., Omata K., Hashimoto Y., Tabata Y., Satoh T. (2010). Alveolar bone tissue engineering using composite scaffolds for drug delivery. Jpn. Dent. Sci. Rev..

[66] Kim M.H., Yun C., Chalisserry E.P., Lee Y.W., Kang H.W., Park S.-H., Jung W.-K., Oh J., Nam S.Y. (2018). Quantitative analysis of the role of nanohydroxyapatite (nHA) on 3D-printed PCL/nHA composite scaffolds. Mater. Lett..

[67] Gómez-Lizárraga K.K., Flores-Morales C., Del Prado-Audelo M.L., Álvarez-Pérez M.A., Piña-Barba M.C., Escobedo C. (2017). Polycaprolactone- and polycaprolactone/ceramic-based 3D-bioplotted porous scaffolds for bone regeneration: A comparative study. Mater. Sci. Eng. C.

[68] Hutmacher D.W., Schantz T., Zein I., Ng K.W., Teoh S.H., Tan K.C. (2001). Mechanical properties and cell cultural response of polycaprolactone scaffolds designed and fabricated via fused deposition modeling. J. Biomed. Mater. Res..

[69] Murphy C.M., O’Brien F.J., Little D.G., Schindeler A. (2013). Cell-scaffold interactions in the bone tissue engineering triad. Eur. Cells Mater..

[70] Zhu M., Li W., Dong X., Yuan X., Midgley A.C., Chang H., Wang Y., Wang H., Wang K., Ma P.X. (2019). In vivo engineered extracellular matrix scaffolds with instructive niches for oriented tissue regeneration. Nat. Commun..

[71] Anselme K., Bigerelle M. (2014). On the relation between surface roughness of metallic substrates and adhesion of human primary bone cells. Scanning.

[72] Chang H.-I., Wang Y., Eberli D. (2011). Cell Responses to Surface and Architecture of Tissue Engineering Scaffolds. Regenerative Medicine and Tissue Engineering—Cells and Biomaterials.

[73] Ferrari M., Cirisano F., Morán M.C. (2019). Mammalian Cell Behavior on Hydrophobic Substrates: Influence of Surface Properties. Colloids Interfaces.

[74] Golub E., Boesze-Battaglia K. (2007). The role of alkaline phosphatase in mineralization. Curr. Opin. Orthop..

[75] Zhou X., Esworthy T., Lee S.-J., Miao S., Cui H., Plesiniak M., Fenniri H., Webster T., Rao R.D., Zhang L.G. (2019). 3D Printed scaffolds with hierarchical biomimetic structure for osteochondral regeneration. Nanomed. Nanotechnol. Biol. Med..

[76] Gregory C.A., Gunn W.G., Peister A., Prockop D.J. (2004). An Alizarin red-based assay of mineralization by adherent cells in culture: Comparison with cetylpyridinium chloride extraction. Anal. Biochem..

[77] Melo P., Tarrant E., Swift T., Townshend A., German M., Ferreira A.M., Gentile P., Dalgarno K. (2019). Short phosphate glass fiber—PLLA composite to promote bone mineralization. Mater. Sci. Eng. C.

